# Wearable Devices for Physical Monitoring of Heart: A Review

**DOI:** 10.3390/bios12050292

**Published:** 2022-05-02

**Authors:** Guillermo Prieto-Avalos, Nancy Aracely Cruz-Ramos, Giner Alor-Hernández, José Luis Sánchez-Cervantes, Lisbeth Rodríguez-Mazahua, Luis Rolando Guarneros-Nolasco

**Affiliations:** 1 Tecnológico Nacional de México/I.T. Orizaba, Av. Oriente 9 No. 852 Col. Emiliano Zapata, Orizaba 94320, Veracruz, Mexico; guillermo.prieto.avalos@gmail.com (G.P.-A.); dci.ncruz@ito-depi.edu.mx (N.A.C.-R.); lrodriguezm@ito-depi.edu.mx (L.R.-M.); luisguarneros@gmail.com (L.R.G.-N.); 2 CONACYT-Tecnológico Nacional de México/I. T. Orizaba, Av. Oriente 9 No. 852 Col. Emiliano Zapata, Orizaba 94320, Veracruz, Mexico; jlsanchez@conacyt.mx

**Keywords:** cardiovascular diseases, monitoring, sensors, wearables

## Abstract

Cardiovascular diseases (CVDs) are the leading cause of death globally. An effective strategy to mitigate the burden of CVDs has been to monitor patients’ biomedical variables during daily activities with wearable technology. Nowadays, technological advance has contributed to wearables technology by reducing the size of the devices, improving the accuracy of sensing biomedical variables to be devices with relatively low energy consumption that can manage security and privacy of the patient’s medical information, have adaptability to any data storage system, and have reasonable costs with regard to the traditional scheme where the patient must go to a hospital for an electrocardiogram, thus contributing a serious option in diagnosis and treatment of CVDs. In this work, we review commercial and noncommercial wearable devices used to monitor CVD biomedical variables. Our main findings revealed that commercial wearables usually include smart wristbands, patches, and smartwatches, and they generally monitor variables such as heart rate, blood oxygen saturation, and electrocardiogram data. Noncommercial wearables focus on monitoring electrocardiogram and photoplethysmography data, and they mostly include accelerometers and smartwatches for detecting atrial fibrillation and heart failure. However, using wearable devices without healthy personal habits will cause disappointing results in the patient’s health.

## 1. Introduction

According to the World Health Organization (WHO) [1], noncommunicable diseases (NCDs) kill 41 million people each year, which is equivalent to 71% of global deaths. Each year, 15 million people between the ages of 30 and 69 die from NCDs, and more than 85% of these premature deaths occur in low- and middle-income countries. Cardiovascular diseases (CVDs) account for the majority of NCD deaths (17.9 million each year), followed by cancer (9.0 million), respiratory diseases (3.9 million), and diabetes (1.6 million). Namely, these four groups of diseases account for more than 80% of premature deaths, as follows: cardiovascular disease (43%), cancer (21%), respiratory diseases (10%), and diabetes (4%).

CVDs are the leading cause of death globally, claiming 17.9 million lives each year. The study of Global Burden of Cardiovascular Diseases and Risk Factors found hypertension to be the most prevalent CVD (65.5%) in 2019, followed by ischemic heart disease (11.4%), peripheral vascular disease (6.5%), cerebrovascular disease or stroke (5.8%), arrhythmias (atrial fibrillation and atrial flutter, 3.5%), rheumatic heart disease (2.3%), heart failure (1.5%), coronary heart disease (1.1%), hypertensive heart disease or cardiopathy (1.08%), congenital heart disease (0.7%), and cardiomyopathies (0.53%) [2]. Such results demonstrate how crucial it is to underpin efforts for hypertension prevention and treatment in order to reduce the global incidence of CVDs.

CVDs affect organs such as the heart and blood vessels, which can cause strokes and coronary and rheumatic diseases. More than 80% of CVD-related deaths are due to coronary heart disease and stroke, and 33% of those deaths occur prematurely in people under the age of 70 [2]. Additionally, unhealthy eating habits, such as having an unbalanced diet and high consumption of salt, sugars, and fats indirectly contribute to the incidence of CVDs, since they promote obesity and overweight. [3]. Preventing premature CVD-related deaths requires identifying people at high risk and ensuring that they receive appropriate treatment. In this sense, access to essential medicines and basic health technologies to treat noncommunicable diseases in all primary healthcare centers is essential to provide CVD treatment and counseling to everyone in need [1,3].

Ministries of health worldwide collaborate to reduce the alarming statistics of global CVD incidence and related deaths. In parallel, wearable technologies are gaining increasing presence in the healthcare sector, as new generations of wearables emerge, driven by the desire of consumers to monitor their own health. Moreover, as new features allow wearable technologies to assess real-time biometric data, their impact on CVD management has become undeniable. The main clinical benefits of using wearable technology to tackle CVD include refining stroke prevention strategies, personalizing atrial fibrillation management, and optimizing the patient–physician relationship. Wearables are changing not only the way clinicians conduct research, but also the future of cardiovascular preventive and therapeutic care [4].

Currently, Android technology is used in 70% of smartphones worldwide, which has resulted in the rising importance of Android application programming [5]. With the advent of new mobile technologies, the mobile application industry is advancing rapidly. There is a variety of operating systems (OSs), such as Symbian OS, iOS, Blackberry, and others, but Android OS is recognized as the most widely used, popular, and user-friendly mobile platform. This open-source Linux kernel-based operating system offers high flexibility due to its customization properties, making it a dominant mobile operating system, which is commonly found implemented in smartphones or wearable devices in the health area [6]. Due to the above, mobile health (mHealth) apps compatible with Android are used for the self-management of CVDs, and there is an increasing trend in their use. The majority of mHealth apps for CVD self-management can provide medical recommendations, medical appointments, reminders, and notifications for CVD monitoring. The main challenges in the use of mHealth apps for CVD self-management include overcoming patient reluctance to use the technology and achieving the interoperability of mHealth applications with different operating systems [7].

The literature reports a substantial number of scientific contributions to CVD management and prevention, including technology development, patient behavior analysis, and monitoring technologies. Researchers such as Lobello et al. [8], Pevnick et al. [9], Akinosun et al. [10], and Ji et al. [11] have proposed legal frameworks based on mobile health (mHealth) technology for the classification, evaluation, and management of resources for monitoring CVD patient health status. On the other hand, authors Hong et al. [12], Lin et al. [13], Sana et al. [14], Dagher et al. [15], Cho et al. [16], Promphet et al. [17], Nasiri et al. [18], Duncker et al. [19], Kinast et al. [20], Chen et al. [21], Khan et al. [22], Wang et al. [23], Scrugli et al. [24], Ramasamy et al. [25], Rai et al. [26], and Hannan et al. [27] reviewed the technological advances in the monitoring of physiological signals from wearable and implantable devices based on flexible and stretchable electronics for CVD monitoring. Joe et al. [28] evaluated the benefits of using wearable devices in adult patients with CVDs. Mizuno et al. [29], Rens et al. [30], Hammond-Haley et al. [31], Xie et al. [32], Ferguson et al. [33], Tobin et al. [34], Nuvvula et al. [35], and Chokshi et al. [36] analyzed physical activity data retrieved from monitoring wearable devices to determine strategies for CVD prevention, monitoring, and control. In turn, Castaneda et al. [37], Shabaan et al. [38], Lou et al. [39], Guo et al. [40], and Tandon et al. [41] developed their own sensors and wearable technologies for CVD care. Nahavandi et al. [42] evaluated the opportunities and challenges of implementing artificial intelligence (AI) techniques in wearable devices. Finally, Reda et al. [43], Surantha et al. [44], Khoshmanesh et al. [45], Santo et al. [46], Akinosun et al. [10], DeVore et al. [47], and Burnham et al. [48] proposed other wearable and mobile innovations to improve CVD care.

When compared to other reviews reported in the literature, our work has four differences. First, we discuss both commercial and noncommercial wearable devices currently available for CVD monitoring. Second, we classify such devices depending on their key features. Then, we identify the most important biomedical variables and sensors used in CVD monitoring. Finally, we discuss the status of the reviewed wearable technologies with respect to FDA (Food and Drug Administration) regulations. The goal of this review is, then, to identify (1) commercial and noncommercial wearable devices currently available for CVD monitoring, (2) the characteristics of such devices, (3) the primary biomedical variables used for monitoring the five most prevalent CVDs (as listed by the Global Burden of Cardiovascular Diseases and Risk Factors Study), and (4) the FDA status of the reviewed wearable technologies.

## 2. Biomedical Variables in CVDs

CVD symptoms vary across people and depend on each condition; however, they usually include irregular heartbeat, high blood pressure, coronary artery–valve damage, and stroke. According to the WHO, more than 17 million people worldwide die from CVDs each year, which is equivalent to half of the deaths that occur in the United States [49]. Around the world, healthcare systems struggle with the rising costs of medical treatment and services; however, remote patient monitoring through wearable devices helps drive down the costs of CVD management and deliver better patient outcomes. In other words, portable and discreet monitoring devices, along with telecommunication technology, are a promising alternative for prompt and accurate medical follow-up of patients with CVDs or those at high risk of developing them [40].

**Figure 1:**
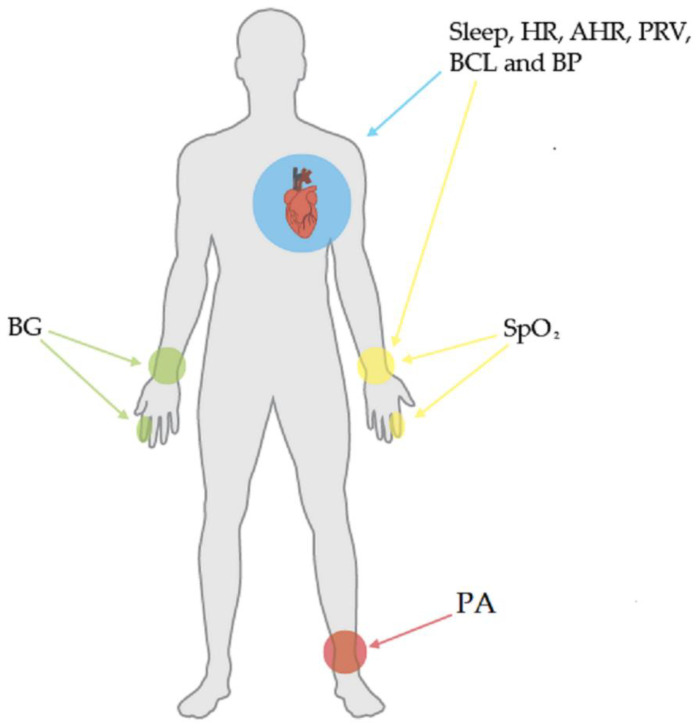
Common biomedical variables and associated body parts.

CVD monitoring using wearable technology involves measuring a series of biomedical variables and patient behaviors, such as lifestyle and eating habits. In the following paragraphs, we briefly explain the biomedical variables involved in CVD monitoring. Subsequently, Figure 1 illustrates the body parts which are commonly associated with these variables.

*Physical Activity* (*PA*). PA benefits the health of every one person, at any age, both men and women. However, the number of people who do not have PA is increasing, and this is mainly due to the sedentary lifestyles that are prevalent today. Most of the world’s population lives in industrialized environments with access to technology, which facilitates tasks that previously required greater physical effort. In addition to the above, the number of leisure offers that do not require moving is increasing; impacting the general health of the world population, reflected in the increasing number of people with health problems such as diabetes, CVD, or cancer [31]. Some of the wearables or sensor technology used to monitor PA are (1) pedometers, which are devices with motion sensors that are usually placed on clothing (usually on the waist) and are intended to record the steps taken during the day. These devices are small, lightweight, nonintrusive, and easy to use. These wearable devices detect movement when walking or running, and the accumulated steps can be displayed digitally on a screen, providing immediate feedback to the user, (2) load transducers, which are used to measure walking activity or held, lifted, or carried loads, and (3) accelerometers which measure energy expenditure, PA intensity, body position, and amount of sleep, based on the measurement of the rate and magnitude with which the body’s center of gravity shifts during movement [50].

*Sleep*. It is a basic human need and is essential for good health, excellent quality of life, and performing well during the day. Several indicators can be used to describe sleep disturbance or sleep disorders. These indicators include, for instance, sleep latency, number and duration of nocturnal awakenings, total sleep time, and repetitive nights of sleep disruption for one week or one month. Both poor sleep quality and short sleep duration are directly associated with CVD incidence. Sleep deficiency can lead to increases in blood pressure and early endothelial dysfunction, which contributes to CVD. Commercial wearable devices, such as the Fitbit Charge Heart Rate and the Oura Ring, aim at monitoring sleep in patients with CVDs through both heart rate and blood pressure [51]. The two sensing technologies most common for monitoring sleep are (1) electroencephalography (EEG), which is a recording of electrical signals from the brain obtained from electrodes placed at different locations on the scalp, and (2) photoplethysmography (PPG), which is a recording of electrical signals representing changes in blood volume in the microvascular bed of the tissue from an optical process. EEG-based systems are the most accurate in identifying all sleep stages relative to PPG-based systems [51].

*Heart rate* (*HR*). It refers to the number of times the heart beats in one minute. Pulse rates vary from person to person and depend on numerous exogenous and endogenous factors, such as age, heart size, gender, genetics, health status, biorhythms, and stress/tension, among others. Wearable devices for HR monitoring are most often used when a patient with CVD performs exercise routines recommended by a health specialist [52]. In the clinical environment, HR is obtained by analyzing electrocardiogram technique (ECG) signals through electrodes attached to the patient’s skin. Challenges that still need to be addressed when using the ECG are avoiding the discomfort and irritation caused by the electrodes and accurately distinguishing between the signal of interest and noise (for example, in seizures). In the fitness industry, HR is assessed during exercise using photoplethysmography (PPG), which is a simple optical technique using low-intensity infrared light to measure blood flow volume. To this end, PPG analyzes the optical reflection of light of different wavelengths that the wearable device (usually a smartwatch) applies to the patient’s wrist or finger [53].

*Average heart rate* (*AHR*). This variable is estimated by counting the number of heartbeats in a given period of time. It is also commonly used in commercial portable devices to assess body condition during physical activity. AHR can be estimated through an ECG using QRS detection algorithms [54,55], whereas for PPG, AHR estimation is generally performed by analyzing the characteristics of the PPG spectrum [56]. AHR is considered a relevant indicator in the diagnosis of CVD [57] and is correlated with problems in the autonomic nervous system and sleep disorders [58]. Recently, from facial thermal imaging and through a machine learning (ML) framework, AHR has been estimated using infrared thermography (IRT). Specifically, it implements support vector regression (SVR) to estimate AHR from features evaluated on the temperature of facial regions of interest (ROIs) [59].

*Pulse rate variability* (*PRV*). It refers to the variations of time intervals between heart beats. Traditionally, PRV is measured as the series of instantaneous cycle intervals obtained from ECG to determine the activity of the autonomic nervous system on cardiac function. PRV is generally measured at rest (sitting or lying down). At rest, in a healthy adult, PRV ranges from 60 to 100 beats per minute. Conversely, during physical activity, PRV oscillates between 150 to 200 beats per minute. Finally, PRV is usually around 60 beats per minute during sleep. Predicting abrupt variations in PRV can help timely diagnosis of CVDs. In fact, some studies claim that PRV may be a more relevant biomarker of CVD than AHR [60]. The technique photochromatography (PCG) is digital highlighting of color change in an object not readily seen by the unaided eye. LYFAS is a biomedical application that, through the optical sensor and LED light of camera to smartphone (Android), can detect PRV by analyzing the blood flow in the index finger of the patient, using the PPG and PCG techniques [61].

*Blood pressure* (*BP*). This biomarker refers to the force that blood exerts against the walls of the arteries. BP can be described with two numbers; that is, x/y in units of millimeters of mercury (mm Hg), where “x” measures the pressure in the arteries when the heart beats and pumps blood through the body, known as systolic pressure. Conversely, “y” refers to the pressure on the artery walls when the heart rests between beats, also known as diastolic pressure. Hypertension is the primary condition associated with increased BP. Overtime, the force or pressure that blood exerts on the artery walls is high enough to cause health problems, such as CVDs. If not treated timely, hypertension can lead to medical conditions such as heart disease, stroke, kidney failure, and eye problems, to name but a few [4]. A potential indicator for estimating BP is the pulse transit time (PTT) it takes to travel through the cardiovascular system from one point to another. This can be calculated from ECG and PPG monitoring of two pulse signals generated by the cardiovascular system [62].

*Blood oxygen saturation* (*SpO_2_*). It indicates the amount of oxygen in red blood cells circulating throughout the human body. SpO_2_ levels for most healthy adults lie in the range of 95% to 100%. A level below this range is indicative that the person needs urgent medical attention, because their organs, tissues, and cells are not receiving the necessary oxygen for their body to function properly [63]. Pulse oximetry (based on the PPG technique) is widely used to measure how much oxygen the blood contains. It is based on the emission of light rays that pass through the blood of the patient’s finger (or earlobe). From the reading of the reflected light rays, the percentage of oxygen in the blood is calculated [64].

*Blood glucose* (*BG*). BG comes from the foods that we consume or is produced by the liver and is found in the bloodstream (it is transported to all cells) and inside the cells (it is transformed into energy). High BG levels are a common indicator of diabetes mellitus, which can cause kidney, neuronal, eye, and cardiovascular diseases. The probability of suffering from these complications increases as the BG level increases and vice versa [65,66,67,68]. BG is measured in milligrams per unit deciliter (mg/dL). Among the noninvasive wearable devices are the wrist-worn ones, based on a system of electrochemically measured glucose concentrations in skin interstitial fluid (ISF) extracted by reverse iontophoresis. Skin ISF surrounds cells and supplies nutrients through diffusion from the capillary endothelium, obtaining a reliable correlation between blood and ISF glucose levels [69].

*Blood cholesterol level* (*BCL*). Cholesterol helps the human body build new cells, insulate nerves, and produce hormones. Normally, the liver makes all the cholesterol that the body needs, but cholesterol also enters the body through animal source foods. Too much cholesterol in blood builds up on the artery walls, narrowing them and slowing or blocking blood flow to the heart muscle. Since blood carries oxygen to the heart, high cholesterol levels may lead to a heart attack, since not enough oxygen is delivered [70]. There are several traditional analysis techniques for cholesterol measurement, such as spectrophotometry, chromatography, and capillary electrophoresis. However, they require a long analysis time, large sample amounts, high cost, and qualified personnel for their implementation, which makes frequent monitoring difficult. In this way, electrochemical biosensors are a serious alternative to overcome some of the disadvantages of the techniques mentioned above, without sacrificing reliability with respect to traditional techniques [71].

*Other biomedical variables*. Variables such as oxidative stress, translational signals, alterations in intracellular calcium management, and mitochondria dysfunction have been proposed as biomarkers of CVDs [72]. However, they are not considered in current commercial wearable devices, which is a potential opportunity for improvement. Below, these additional biomedical variables are briefly described.

*Oxidative stress in cardiac disease*. This happens when compounds that are not useful for their optimal functioning (hydrogen peroxide, free radicals, etc.) are produced in the human body. If these compounds reach excessive levels in the body, the functionality of their membranes breaks and the cells die, resulting in cardiac dysfunction. In a healthy individual with a proper diet and lifestyle, oxidative stress can be minimized; not completely, but it can be controlled [73].

*Cell signaling in the cardiovascular system*. The cellular elements of the heart and the vascular wall have a series of particular receptors and a complex intracellular mechanism that controls the appropriate responses to extracellular stimuli, that, in some cases, alters the functions of the cells in the heart and the vascular wall, causing pathological situations such as cardiovascular disorders [74].

*Abnormalities in intracellular calcium handling*. The human body needs calcium (Ca), which is a mineral used to build and maintain its bones optimally in addition to performing other functions. Ca is the most abundant mineral in the body, and most is stored in the bones and teeth, which gives them structure and rigidity. Ca regulates various functions in the human body, including heartbeat, muscle contraction, and neuronal synapses. The weakening of cardiac contraction and the propensity for arrhythmias are related to an imbalance of calcium in cardiomyocytes (mainly in older adults) [75].

*Mitochondrial dysfunction*. This dysfunction is caused by a defect in energy production within the cells of an organism. Energy is produced within organelles contained in cells called mitochondria. All living beings need energy for their metabolism to work; for example, to grow, move, and think, among others. This dysfunction participates in the pathology of different diseases, such as neurodegenerative and cardiovascular [76].

## 3. Methods

This paper is a review of sensor technologies from the IoT perspective to determine whether it is possible to monitor specific diseases, namely CVDs, using wearable devices and provide remote healthcare to older adults. The review follows the guidelines of the PRISMA [77] statement to ensure the proper organization and clarity of its results.

**Inclusion and exclusion criteria.** We initially obtained 24,900 search results from all the databases; yet, to refine the search, we deleted 632 results of works published before 2010, leaving 24,268 potential sources. Below, we describe the inclusion and exclusion criteria followed in this review.

*Inclusion criteria:* The review comprises research works related to (1) wearable technologies for CVD monitoring, (2) wearable devices for CVD monitoring, (3) commercial and noncommercial wearable devices for CVD monitoring, and (4) FDA-approved medical devices published from 2010 to 2021.

*Exclusion criteria:* We discarded (1) sources not written in English, (2) non-peer-reviewed sources, (3) letters and reports, (4) conference and symposium proceedings, (5) and non-primary studies.

**Information Sources.** We grouped the keywords in our research questions into two categories or knowledge areas—healthcare and computing technology—to determine the databases for the review. In terms of healthcare, the search was conducted on AHA Journals, Annual Reviews, BioMed Central, Clinical Trials, JMIR, Medline Plus, and PubMed. Conversely, computing technology databases included the digital libraries of Hindawi, IEEE Xplore, Inderscience, IOP science, JACC, MDPI, Nature, Science Direct, Springer Link, and Wiley Online Library.

**Search Strategy.** The search strategy used in this review was to combine keywords with Boolean connectors to limit the search results. The search keywords were considered from the key concepts that are part of the research questions that led to answering them. Intermediate searches were ordered to find the search terms to use in subsequent queries:

1. Main CVDs worldwide.
2. Biomedical variables of diagnosed CVDs.
3. Wearable devices used to measure these biomedical variables.
4. Sensor-based wearable devices available in the market.
5. Commercial and noncommercial wearable devices for CVD monitoring.
6. FDA-approved commercial wearable devices.

Progressively, the results obtained from each query listed above contained new terms that were relevant to this study.

**Figure 2:**
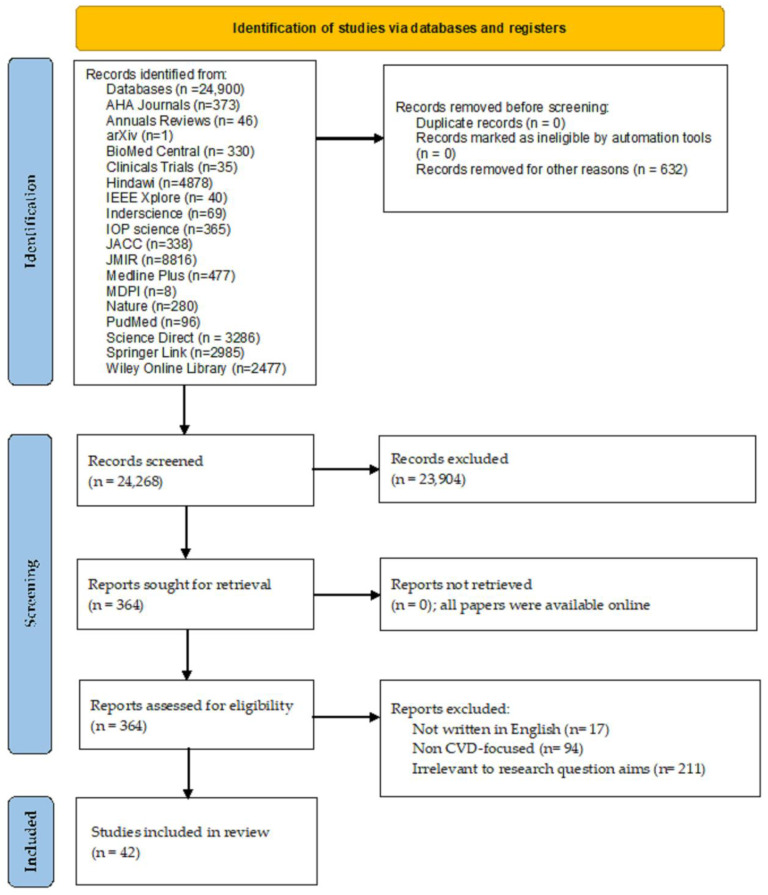
PRISMA flow diagram of the search strategy.

**Selection process.** Initially, we identified 24,268 relevant papers based on their title and abstract. Then, three subject matter experts (SMEs) screened each work and organized the data into seven categories: device brand, device model, device type, targeted CVD, device functionality, used sensors, and FDA status. Following the SME analysis, we discarded 23,904 publications. Then, we thoroughly reviewed the 364 works left for further analysis in terms of their research goals and questions. Finally, only 42 of these studies were considered to comply with all the inclusion criteria. Figure 2 introduces the PRISMA-based diagram of our search strategy.

The selected 42 studies were downloaded in full text from their corresponding databases: Science Direct (20), NIH (3), Wiley Online Library (3), arXiv (2), AHA Journals (2), IOP science (2), JMIR (2), Springer Link (2), Annual Reviews (1), BioMed Central (1), IEEE Xplore (1), JACC (1), MDPI (1), and Nature (1).

**Data collection and analysis.** We used structured tables to organize the information collected during the review of the 42 primary studies. Three SMEs oversaw the analysis to extract relevant information on current commercial and noncommercial wearable devices used for monitoring physiological variables in patients with CVDs. Information of interest included targeted CVD, wearable device brand, model, and type, key device features, device operating mechanism, used sensor(s), and FDA status.

## 4. Results

### 4.1. Study Selection

Our initial search for relevant papers yielded 24,900 results. Figure 2 summarizes the distribution of such results by their source database. Then, we removed 632 records from further analysis. The resulting 24,268 records were screened for relevance based on their title and abstract; then, 23,904 records were subsequently removed. The remaining 364 records were assessed for eligibility by performing a full-text analysis. The assessment revealed that 322 records were ineligible according to our exclusion criteria: (i) non-English-language research, (ii) non-CVD-focused research, and (iii) research irrelevant to the research goal. Finally, the review comprised 42 records left after applying the inclusion and exclusion criteria for eligibility.

### 4.2. Study Characteristics

This review analyzes both commercial and noncommercial wearable devices for CVD monitoring. Commercial wearable devices comprise both presale devices and those available in the market by the time of writing this paper. Conversely, prototype devices and research devices were classified as noncommercial. In total, we found 31 commercial and 32 noncommercial wearable devices for CVD monitoring.

## 5. Commercial Wearable Devices

A substantial number of companies around the world market portable devices that conveniently help CVD patients monitor biomedical variables in their day-to-day life. However, not all of these devices have been approved by regulatory bodies. The US Food and Drug Administration (FDA) regulates the sale of medical devices and provides consumers with assurance that, once such devices are commercialized, they are safe and effective in their intended use. In other words, the FDA is responsible for protecting public health by ensuring that commercial medical devices meet a series of requirements. Table 1 summarizes our findings with respect to the most important elements of each reviewed device, including their FDA status.

**Figure 3:**
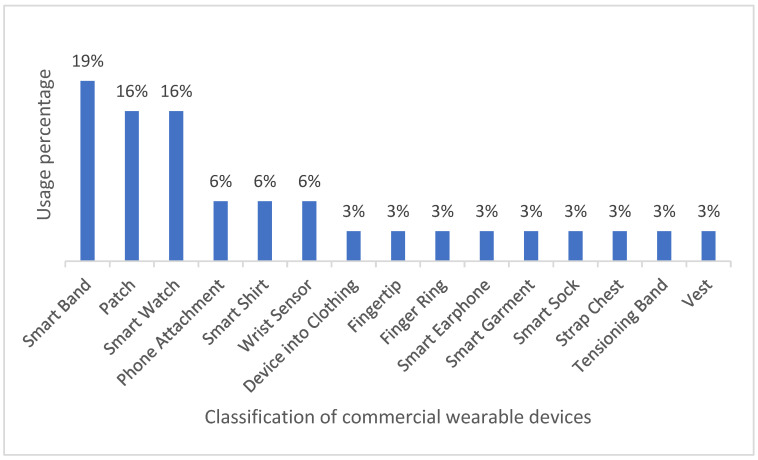
Classification of commercial wearable devices.

Most of the wearable devices commercially available for CVD monitoring are smartwatches and wristbands (35% of occurrence), whereas the least common devices include finger rings, vests, and devices attached to clothing (3% each). On the other hand, only 16% of the reviewed works use patches for monitoring CVD biomedical variables, even though the heart is the most affected organ in CVDs. Such results reveal a growing interest in sensor-based technologies, which can be conveniently and easily implemented in wearables such as wristbands and smartwatches. In fact, consumers usually prefer these devices to patches, since they can be comfortably worn and rarely interfere with day-to-day activities. Figure 3 illustrates the categories of wearable devices found in the review.

**Figure 4:**
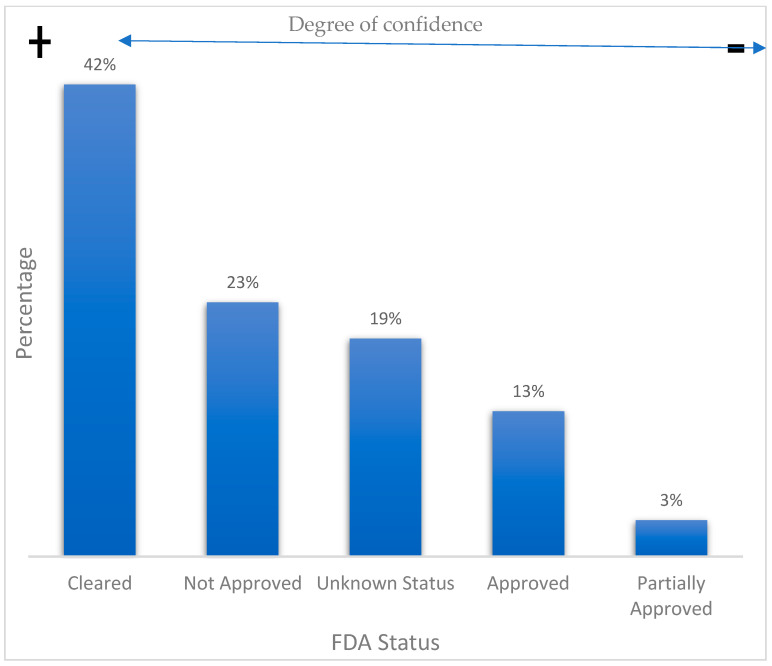
FDA status of commercial wearable devices for CVD monitoring.

With regard to device FDA status, we considered five categories: approved, partially approved, cleared, unapproved, and unknown. Both approved and partially approved status mean that the benefits of the wearable device outweigh its known risks for intended use. On the other hand, a cleared FDA status implies that the device manufacturer can demonstrate that their product is substantially equivalent to another legally marketed device. An unapproved status conveys that the FDA does not authorize the use of the device. Finally, an unknown status implies that we could not find information regarding the FDA status of a device. In this sense, it is worth mentioning that many commercial devices do not disclose public information on their FDA status. According to our findings, 58% of the reviewed commercial devices do have some FDA approval, whereas the remaining 42% are commercially available but lack FDA registration or approval. On the other hand, we were unable to find further FDA information on 19% of the reviewed commercial wearables. Figure 4 summarizes these results.

One of the main objectives of our review was to determine which biomedical variables are commonly monitored by wearable technologies for CVD care. In this sense, we found that wearable devices can usually read more than one type of biomedical variable. Such results were documented, and a summary of them is listed in Table 2.

**Figure 5:**
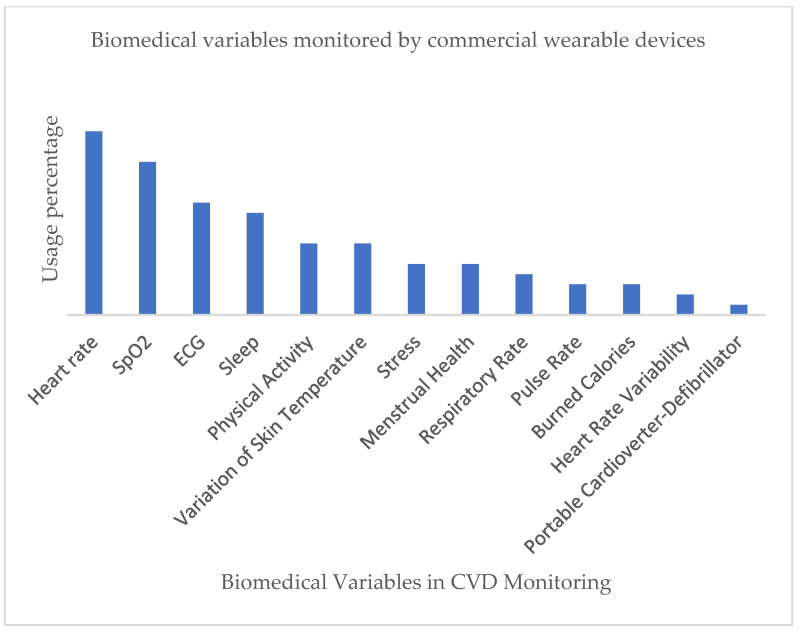
Classification of commercial wearables for CVD monitoring with respect to biomedical variables.

Figure 5 below is a graphical representation of the results summarized in Table 2.

As shown in Figure 5, the main biomedical variables in CVD monitoring include HR, SpO_2_, and ECG. Rapid scientific progress and modern technology have made it possible for companies to fabricate increasingly compact monitoring solutions that can be easily built in wearable items. In parallel, wearable technologies have turned into a relatively affordable consumer trend that allows for convenient self-health monitoring during day-to-day activities. However, a major challenge of commercial wearable devices remains to improve the monitoring performance and unobtrusive quality of such devices, regardless of their design and aesthetic characteristics.

## 6. Noncommercial Wearable Devices

Our review of noncommercial wearable devices for CVD monitoring comprises both prototype devices and those devices developed solely for research purposes. Generally, prototype devices are primarily designed to assess their viability for monitoring the health status of patients with CVD. Our findings regarding noncommercial wearable technologies for CVD monitoring are organized in Table 3, which summarizes the following information:

- Research year of publication.
- Type of CVD that can be monitored.
- Type of wearable device (e.g., smartwatch and wristband).
- Brief description of the research contribution.
- Sensors or technology used for CVD monitoring.
- Device real-time monitoring capabilities.

**Figure 6:**
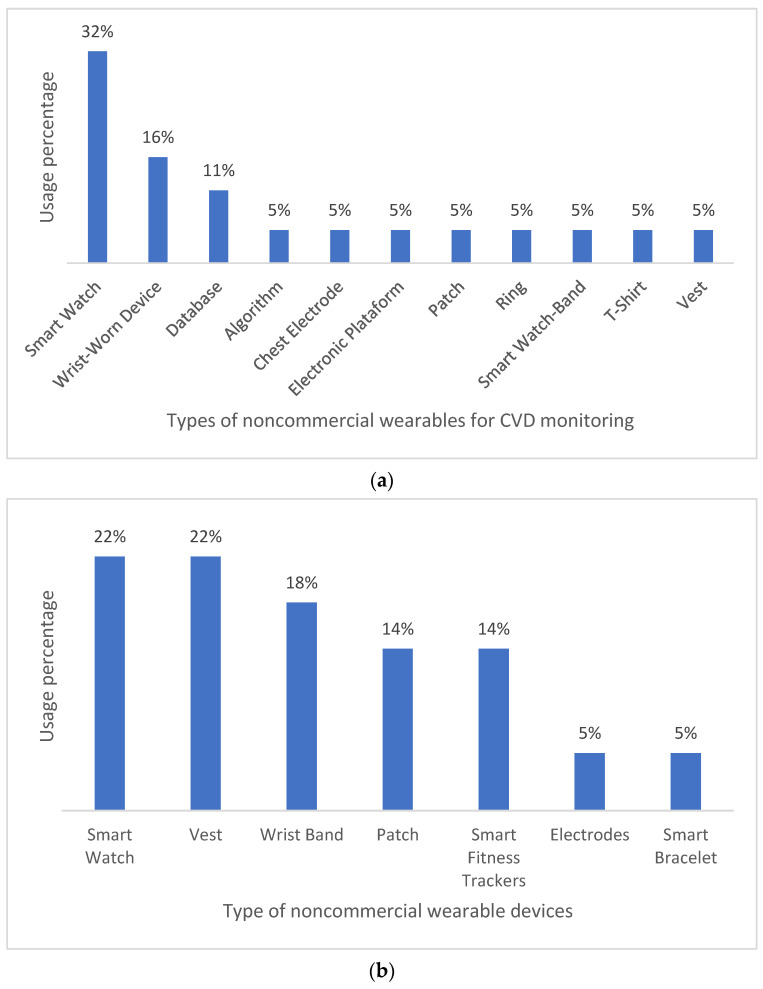
Types of noncommercial wearables for CVD monitoring—(**a**) atrial fibrillation and (**b**) heart failure.

When compared to commercial wearable devices, noncommercial wearable technologies are generally developed for arrhythmia and heart failure monitoring. Wrist-worn devices such as smartwatches and smart wristbands are the most common wearable technologies in these cases (53% in arrhythmia monitoring and 59% in heart-failure monitoring). Conversely, less common technologies include patches, rings, t-shirts, and vests for arrhythmia monitoring, and vests, patches, and electrodes for heart-failure monitoring. Wrist-worn devices are usually preferred to other wearable alternatives due to a relatively high comfort–monitoring accuracy ratio. Figure 6 below graphically summarizes our findings.

Monitoring patients with CVD in real time allows healthcare professionals and patients themselves to receive continuous, live health status updates during daily activities. Table 4 summarizes our findings with respect to noncommercial devices with real-time monitoring capabilities.

Finally, Table 5 summarizes our findings with respect to the sensors and technologies commonly implemented in noncommercial wearables for CVD monitoring. As can be observed, accelerometers, ECG, and PPG are preferred over other alternatives, such as neural networks, and they are especially implemented in wrist-worn devices. Once again, the good comfort–accuracy ratio of such devices contributes to their popularity.

This review provides insightful information on wearable health monitoring solutions for patients with CVDs. First, our findings reveal that commercial wearable technologies generally focus on measuring HR (20%), SpO2 (16%), and ECG (12%) as biomedical variables. Measurements are usually obtained via sensors or compact technologies easily integrated in the wearables. Additionally, the most commercial wearables for CVD monitoring include smart wristbands (19%), smartwatches (16%), and wrist sensors (6%). As for noncommercial wearables, most of them are wristbands. In addition, noncommercial wearables generally rely on accelerometers, PPG, and ECG (72%) for CVD monitoring.

Second, in both commercial and noncommercial wearables, wrist-worn devices are preferred over other items and clothing (e.g., vests, rings, and T-shirts), since they are comfortable and unobtrusive to patients. Nevertheless, ongoing research is exploring how to implement health monitoring technology into daily wear garments in a way that is just as convenient, affordable, and comfortable to consumers as wearing wrist-worn devices. However, these garments, of a shirt or dress type, for instance, must have certain characteristics to be as convenient, safe, and effective as wrist-worn devices. Some of these features are data reading accuracy, real-time wireless monitoring, water resistance, data security, and portability. Overall, we found that commercial CVD monitoring garments are significantly less common (21%) than their noncommercial counterparts (41%).

## 7. Discussion

### 7.1. Challenges and Trends

Wearable technologies for CVD monitoring significantly help drive down the costs of in-hospital treatments. In terms of continuous and outpatient monitoring, smart wearables improve diagnosis precision, thus providing patients with convenient solutions for self-care through the ongoing monitoring of biomedical variables during daily routines. CVD monitoring techniques greatly vary, and their usability depends mostly on the type of condition to be prevented or managed and the type of monitoring. For instance, ECG and PPG are generally implemented for continuous and ambulatory monitoring in fitness bands and smartwatches. ECG is a test widely recommended in the follow-up of some heart diseases. It involves the recording of heart electrical activity through the surface of the patient’s body. Then, based on the difference in electrical potential between two distant points of the body, ECG can measure a series of biomedical variables to detect heart diseases such as heart failure and arrhythmias. However, ECG technology is not recommended for monitoring the risk of heart attack. In this case, conventional electrocardiography with electrodes remains the best option.

PPG is another technique for monitoring CVD biomedical variables. It involves injecting photons into human body tissue and analyzing the reflected light. PPG has proved to yield better results when performed at the wrist level via bands or smartwatches, which are unobtrusive devices that patients can conveniently carry with them during day-to-day activities. As its main setback, PPG has little robustness and reliability during physical activity or when the patient is in motion.

In addition to ECG and PPG, other techniques are used to monitor important heartbeat information, such as ballistocardiography (BCG) and phonocardiography (PCG). BCG is a technique used to measure ballistic forces generated by the heart. The technique produces a graphical representation of repetitive movements of the human body that arise as blood is suddenly ejected into the great vessels with each heartbeat. BCG is usually integrated into smart wearables using highly sensitive accelerometers connected to the patient’s body surface, usually the torso. In turn, PCG technology can be used to record heart sounds. Unfortunately, since both BCG and PCG are sensitive to ambient noise, it is unlikely that we will find them integrated into current wearable devices.

The skin covers most of the human body, so it serves as an optimal mode for noninvasive wearable devices for medical care. Skin-based wearable devices can be used for physiological and psychological monitoring for the treatment of different diseases, for example, CVDs. In addition, it can also be used for the diagnosis of different diseases through the qualitative and quantitative analysis of skin secretions, such as sweat. In the case of epidermal wearable devices, they imply the direct union to the skin, such as a tattoo, generally known as electronic skin (e-skin). E-skin is made with flexible electronic components, such as conductive links (e.g., liquid metal alloys for printing ultrathin circuits, graphene, gold nanorods, or various polymers with a rubber backing), allowing each patient to be managed as a separate database of medical information, which is relevant to the medical staff caring for them. When an e-skin is inserted into the patient’s skin, it can record information about the patient’s biomedical variables through its small electrodes and simultaneously send such data to smartphones or other connected devices. In addition, e-skin can receive energy from the electrophysiological processes of the human body, making it possible to work without batteries. E-skin adapts to any shape and even works if bent, twisted, or stretched, since it has properties similar to those of light fabrics; that is, e-skin adapts to the flexibility of the human body, which is an advantage over conventional wearable devices. This noninvasive medical technology could allow healthcare experts to monitor and diagnose arrhythmia problems, heart activities of premature babies, sleep disorders, and brain activity, among other diseases.

On the other hand, it is estimated that more than one billion people around the world have hypertension or elevated BP. Of that 1 billion, two-thirds are in developing countries that also lack adequate sanitary facilities. Daily monitoring of BP is vital for these patients since hypertension is often asymptomatic. Due to the lack of monitoring, hypertension is one of the main causes of premature death around the world. The precise measurement of the PA requires trained medical personnel; therefore the development of the e-skin adds to the challenge of meeting the objective of WHO to reduce hypertension by 25% by 2025. As healthcare technology becomes smaller and smarter, wearable devices such as e-skin could minimize ways in which they interfere with a patient’s daily life.

Currently, e-skin continues to be improved through research and development work. Moreover, the trend in its development indicates that it could be more reliable and accurate and less invasive than traditional methods. Therefore, e-skin will strengthen confidence in consumers who are considering using it for self-care in health.

### 7.2. Emerging Solutions

As wearable trends progressively grow and improve, they begin to shape healthcare, along with current technological developments in hardware and software. Additionally, trends in the Internet of Things (IoT) and AI have become key allies in the development of mHealth wearables. AI techniques and neural networks are implemented into wearables (through signal processing techniques and deep learning) to overcome current technological barriers and improve the reliability of ambulatory monitoring systems and the accuracy of ECG and PPG signals. Overall, both AI and neural networks increase the performance of wearable devices and the accuracy of measurements of CVD biomedical variables. Hence, as the reliability and accessibility of wearable devices increases simultaneously, their acceptance also increases among consumers. Wearables are becoming increasingly convenient solutions for continuous ambulatory monitoring of CVD during day-to-day activities.

The different sensors that make up mHealth wearables collect daily routine data that interact with technological platforms. In this sense, the IoT is a promising alternative for managing data provided by wearable devices. The IoT paradigm can generate medical information and critical event alerts that can be shared with health specialists and used to interact with social networks. A major challenge in the IoT paradigm, however, remains to find the best practices for handling confidential patient information. In this sense, multiple data privacy service providers are already working on it.

Finally, CVD monitoring technologies can also be integrated in smartphones through device cameras or accelerometers. The main issue in this case is that not all smartphones have the same level of precision in terms of recording biomedical variables. Since this is a device function provided at the design level of the mobile device, it certainly goes hand in hand with the cost of the device.

### 7.3. Limitations

This review of commercial and noncommercial wearables for CVD monitoring has four main limitations. First, we did not take into account clinical scenarios or comparative studies assessing the quality of life of patients using wearables for CVD monitoring. Although remote healthcare technology is a current trend, certain usability factors remain to be further studied and improved before wearable technologies become widely accepted. Second, this review does not analyze the mobile applications associated with the reviewed wearable devices. Third, we did not review studies on consumer acceptance of wearables for CVD monitoring. Finally, FDA status information was not available for many of the reviewed devices. Unfortunately, CVD monitoring wearables lacking FDA approval may not have the opportunity to achieve high-quality remote healthcare.

## 8. Conclusions

This review found that wearable devices for remote CVD monitoring rely on sensors/biosensors to obtain accurate measurements of relevant biomedical variables. As measured by these wearable devices, the most critical biomarkers of CVD include heart rate, oxygen saturation, and ECG. Consequently, only wearable devices with suitable sensors that are coordinated by computer applications can provide medical data relevant to CVD monitoring. The use of a given type of wearable over another type greatly depends on the condition to be monitored; however, overall, the wearables most frequently cited in the literature include wristbands (19%), smartwatches (16%), and patches. It remains uncertain whether wearable devices with built-in biosensors can be used for timely detection of lethal diseases, such as malignant neoplasms (various types of cancer). Once this question is resolved, it would be important to analyze the developmental stage of such devices for their broad use as a final product.

In addition, this review analyzes the medical-grade precision and reliability of the cited wearables by listing their FDA status. In this sense, we found that 13% of the cited devices were FDA-approved, 3% were partially approved, 42% were FDA-cleared, 23% were unapproved, and 19% had an unknown FDA status.

The scope of this research is limited to wearable biomedical devices that allow for CVD monitoring via the measurement and assessment of biomedical variables. Our main findings can be summarized as follows: 35% of the cited commercial devices are either smart wristbands or smartwatches, and 58% hold some FDA status (approved, partially approved, cleared). FDA-approved wearables monitor either AF, heart rate, or both. The most common biomedical variables for CVD monitoring are HR (20%), SpO2 (16%), and ECG (12%). Finally, most noncommercial wearable devices for CVD monitoring are smart wristbands, smartwatches, and patches.

## Figures and Tables

**Table 1:** Commercial Wearable Devices for CVD Monitoring.

Device Type	Device Brand	Device Model	Monitoring Features	Sensors Used	FDA Status/Year/AP (AP: Accuracy Percentage)	Android Compatibility
Smartwatch	Withings	Move ECG [78]	Records ECG readings with or without a phone nearby, as the data can be stored on the watch until the next sync.	ECG with 3 electrodes, altimeter, and accelerometer.	Cleared/2021/98.1%	Yes
	Fitbit	Versa 2™ [79]	Monitors and records patient physical activity. Analyzes sleep phases, SpO_2_, variation of skin temperature, respiratory rate, quality of sleep. Records burned calories, menstrual health, stress, moods, guided breathing sessions, HR and resting HR, and cardiovascular fitness.	Three-axis accelerometer, optical HR monitor, infrared, and red-light sensors for SpO_2_ monitoring, altimeter, vibration motor, NFC, ambient light sensor, Wi-Fi antenna (802.11 b/g/n), microphone, device temperature sensor (detection of variations in skin temperature only available for Premium users).	Cleared/2020/ 50% (for steps counter)	Yes
	OMRON	Heart Guide [80]	It is a portable BP monitor.	Accelerometer, PPG HR, oscillometric BP monitor.	Approved/2019/ 94%	Yes
	Apple	Watch Series 7 [81]	Reads blood oxygen levels. Monitors HR and PA. Records sleep hours, among others.	Blood oxygen sensors, electric HR sensor, optical HR sensor, S7 SiP Dual Core Chip, digital crown with haptic feedback, GPS, compass, altimeter, horn, and microphone.	ECG approved/2018, oximeter not approved/ 98% (for ECG)	No
	Huawei	Band 6 [82]	Monitors HR 24/7, day and night SpO_2_. Tracks menstrual cycle, sleep, and stress.	Accelerometer sensor, gyroscope sensor, optical heart rate sensor.	Not approved/not applicable/ not available	Yes
Smart Bracelet	MOCACARE	MOCACuff [83]	Monitors HR and BP. Categorizes BP levels with a color-coded indicator system that correspond to the American Heart Association (AHA) categories.	Information not available.	Approved/2017/ 95% (for HR and BP)	Yes
	Fitbit	Charge 4 [84]	Monitors and records patient physical activity. Analyzes sleep phases, SpO_2_, variation of skin temperature, respiratory rate, quality of sleep. Records burned calories, menstrual health, stress, moods, guided breathing sessions, HR and resting HR, and cardiovascular fitness.	Three-axis accelerometer, optical HR monitor, GPS + GLONASS, infrared and red-light sensors for SpO_2_ monitoring, device temperature sensor (detection of variations in skin temperature available in the Fitbit app), vibration motor, NFC (near field communication) chip, altimeter.	Not approved/not applicable/ 50% (for steps counter)	Yes
	BIOSTRAP	Armband HRM [85]	Provides biometric information, such as HR and deep sleep through a clinical grade pulse oximeter.	Armband heart rate sensor–optical HR technology that accurately measures HR, burned calories, traveled distance, speed, and pace.	Not approved/not applicable/ not available	Yes
	Xiaomi	Mi Smart Band 5 [86]	Monitors HR (full-day HR, manual HR, resting HR, and HR curve) and sleep (seep sleep, light sleep, rapid eye movement (REM), naps). Tracks women’s health (provides recordings and reminders of menstrual cycle and ovulation phases). Monitors stress (breathing exercises, inactivity alerts, step counter, goal setting).	Six-axis sensors: 3-axis low-power accelerometer and 3-axis gyroscope, PPG heart rate sensor, and microphone.	Unknown/ not applicable/ 70% (for sleep tracking)	Yes
Smart Band	HEALBE	GoBe3 [87]	Monitors HR and arterial BP. automatically tracks calorie intake, body hydration, and stress levels.	Bioimpedance sensor, accelerometer, piezoelectric sensor, and galvanic skin response sensor.	Unknown/ not applicable/ not available	Yes
	ViSi Mobile	The ViSi Mobile System [88]	Monitors HR, pulse rate, respiratory rate, BP, SpO_2_, body temperature. Detects arrhythmia, falls, and posture.	Information not available.	Approved/2013 /not available	Yes
Wrist-Sensor	Oxitone	Oxitone 1000M [89]	Measures SpO_2_, skin temperature, pulse rate variability, respiratory rate. Detects falls, steps, and motion.	Skin temperature sensor.	Cleared/2017/97% (for SpO_2_)	No
	VinCense	Wireless Health Monitoring System (whms) [90]	It is a wireless health monitoring system for pulse rate, SpO_2_, respiratory rate, and skin temperature.	Information not available.	Unknown/ not applicable/ 99% (for skin temperature)	Yes
Smart Clothes	Zoll^®^ (Vest)	LifeVest^®^ [91]	Portable automatic defibrillator that stabilizes heart rhythms through an electrical discharge in the chest (in the heart) of the patient.	Garment, electrode belt, and monitor.	Approved/2019/ 92%	No
	Hexoskin (Smart Shirt)	Astroskin [92]	Performs continuous monitoring (48 h) of BP, blood oxygenation, 3-track ECG, breathing rate, skin temperature and physical activity.	ECG, accelerometer, temperature sensor.	Not approved/not applicable/ not available	Yes
	Sleeplay (Smart Sock)	Owlet Smart Sock 3 Baby Monitor [93]	Monitors the baby’s HR and oxygen level during sleep. It is tracked wirelessly via Bluetooth.	Optical HR sensor.	Not approved/ not applicable/ 89% (for oxygen level)	Yes
	Spire Health Tag	Spire [94]	Monitors stress levels, sleep, HR, and breathing patterns. It can be placed on clothes; it is hypoallergenic and water resistant.	Capnographer, ECG and accelerometers.	Not approved/ not applicable/ not available	Yes
	Vivometrics (Smart Shirt)	The LifeShirt system [95]	Records BP and HR to later send the records to a health professional for medical diagnosis.	Monitor respiration, activity and posture, ECG.	Cleared/2005/ not available	Yes
	HealthWatch Technologies (Smart Garment)	Master Caution^®^ [96]	Monitors cardiac ischemia, arrhythmias, respiration, vital signs. Detects falls, inactivity, and skin temperature. Can be used both inside and outside hospital settings.	3–15 lead ECG monitoring. The garment is the sensor.	Cleared/2015/ not available	Yes
	Medtronic (Strap Chest)	Zephyr [97]	Monitors HR.	ECG.	Cleared/2010/ not available	Yes
Patch	iRhythm	Zio^®^ [98]	Records ECG data as the patch is attached to the chest. Records up to 14 days of electrical activity of the heart during daily activities. Once the monitoring is complete, the patch is sent to the treating physician to extract and value the recorded data. The patch can also detect irregular heart rhythms, such as arrhythmia.	ECG.	Cleared/2021/ 99% (for arrhythmia)	Yes
	Preventice	BodyGuardian^®^ Heart [99]	Small wireless heart activity monitor that adheres to the chest via a disposable strip. The strip can be repositioned as needed thanks to its medical-grade adhesive and electrode gel and should be replaced periodically during the monitoring period. The monitor is returned to the service provider.	Accelerometer, ECG.	Cleared/2012/ not available	No
	BioTelemetry	BioTel Heart’s MCOT Patch MCOT: Mobile Cardiac Outpatient Telemetry [100]	Monitors, detects, and transmits abnormal heart rhythms wirelessly. It has been shown to detect atrial fibrillation (≥30 s) with a sensitivity and positive prediction of 100%.	Accelerometer, ECG.	Cleared/2016/ 100% (for atrial fibrillation)	No
	Wellysis	S-Patch Cardio [101]	Records ECG data to assess supraventricular and ventricular arrhythmias.	Accelerometer, ECG.	Unknown/ not applicable/ 95%	Yes
	VitalConnect	Vital Patch [102]	Monitors cardiac function. Sends patient data to a secure cloud for real-time monitoring of different cardiac arrhythmias.	Accelerometer, ECG, thermistor.	Cleared/2017/ 59.2%	Yes
Phone Attachment	AliveCor^®^	KardiaMobile [103]	It can associate cardiac and respiratory symptoms (atrial fibrillation, sinus bradycardia, sinus tachycardia, and arrhythmia) through its ECG patterns.	Mobile electrode with a built-in ECG.	Cleared/2014/ 94% (for arrhythmia)	Yes
	PAI (personal activity intelligence)	PAI Health [104]	The PAI software records the patient HR to optimally manage their health.	Information not available.	Unknown/ not applicable/ not available	No
Finger Ring	Oura	Oura Ring [105]	Based on body temperature, HR, and HR variability (HRV), it records relevant data on monthly menstrual periods, physical activity, and sleep periods.	Body temperature sensor, optical, infrared sensors, and a 3D accelerometer and gyroscope.	Not approved/ not applicable/ 99.9% (for HR) and 98.4% (for HRV)	Yes
Fingertip	iHealth	Pulse Oximeter iHealth Fingertip [106]	Offers an affordable, reliable, and accurate way to check pulse and SpO_2_ levels.	Optical sensor: red light (wavelength is 660 nm, 6.65 mW), infrared (wavelength is 880 nm, 6.75 mW).	Cleared/2013/ 99%	Yes
Smart Earphones	FreeWavz	FreeWavz-Blue [107]	Wireless smart earphones with built-in sensors for HR and fitness monitoring.	Three-axis accelerometer, two electret condenser microphones on each side, pulse oximeter.	Unknown/ not applicable/ not available	No
Tensioning Band on the Arm.	iHealth	Tensiometer Bras iHealth Track (KN-550BT) [108]	Monitors Pulse and BP.	BP sensor.	Cleared/2016/ not available	Yes

**Table 2:** CVD biomedical variables monitored by commercial wearable technology.

Biomedical Variables	FDA Devices	Non-FDA Devices	Total
HR	9	9	18
SpO_2_	7	8	15
ECG	9	2	11
Sleep	3	7	10
Physical Activity	2	5	7
Skin Temperature	5	2	7
Menstrual Health	1	4	5
Stress	1	4	5
Respiratory Rate	0	4	4
Burned Calories	2	1	3
Pulse Rate	2	1	3
HRV	2	0	2
Portable Cardioverter-Defibrillator	1	0	1

**Table 3:** Noncommercial/research wearables and sensors for CVD monitoring.

CVD Type	Device Type	Research Description	Sensors or Technology Used	Real-Time Monitoring
Atrial fibrillation	Massachusetts Institute of Technology (MIT)–Beth Israel Hospital (BIH) Atrial Fibrillation database and the MIT–BIH Arrhythmia database were used as training data and verified the algorithm performance. (2020)	Evaluates an inexpensive heart rate monitor (i.e., a chest patch) with a machine learning algorithm (MLA) capable of accurately detecting AF. The monitor can also transmit ECG data that could be used to confirm AF [109].	Detection algorithm using a decorrelated Lorenz plot.	No
	Ring and pulse oximeter (2020)	Evaluates the performance of a wearable ring-type device for detecting AF using deep learning analysis of PPG signals obtained from the patient [110].	PPG and deep learning.	Yes
	Smartwatch (2019)	Evaluates the ability of a commercial smartwatch, the AliveCor KardiaBand (KB), to detect atrial fibrillation (AF) or sinus rhythm in comparison with the 12-track electrocardiogram (ECG), obtaining results that demonstrate a moderate diagnostic accuracy [111].	ECG.	Yes
	Smart watchband (2019)	Evaluates the accuracy of PPG technology in heart rate monitoring for diagnosing AF in comparison with conventional electrocardiography [112].	PPG.	Yes
	T-shirt (2019)	Discusses the development of a portable device for community screening of asymptomatic AF using a wireless ECG worn on a T-shirt [113].	ECG.	Yes
	Smartwatch (2019)	Discusses evidence on the performance smartwatches in terms of AF detection, concluding that it is still premature to consider them as a first option; however, the future certainly looks encouraging [114].	PPG and ECG.	Yes
	Smartwatch (2019)	Compares the accuracy between recordings from an insertable cardiac monitor (ICM; Reveal LINQ) and a watch with AF detection (AFSW; Apple Watch with Kardia Band), concluding that an AFSW is sensitive in detecting AF, is an inexpensive option, and is not invasive therapy in the long-term follow-up and treatment of AF [115].	Smart Rhythm 2.0, a convolutional neural network.	Yes
	Algorithm (2019)	Discusses the development of an algorithm that accurately detects AF using the PPG technique when the patient performs daily activities [116].	PPG.	Yes
	Chest electrodes (2019)	Discusses the development of a high-precision portable ECG device optimized for AF detection [117].	Prototype ECG.	Yes
	Wrist-worn device (2018)	Proposes an AF detection algorithm using the PPG technique implemented in a self-designed wrist-worn device [118].	PPG.	Yes
	Smartwatch (2018)	Evaluates the accuracy of the AliveCor KardiaBand (KB) with respect to a 12-lead ECG in detecting AF from sinus arrhythmia [119].	ECG.	Yes
	Patch (2018)	Evaluates a patch with an integrated ECG for AF detection, concluding that individuals monitored by this means had an opportunity to receive care earlier if AF was detected, if compared with unmonitored controls [120].	ECG.	Yes
	Wrist-worn prototype fitness tracker device (2018)	Evaluates a convolutional recurrent neural network with applications in PPG-based AF diagnosis [121].	Convolutional–recurrent neural network architecture and PPG.	Yes
	Smart watchband (2018)	Reviews available portable technologies to determine their potential advantages and disadvantages in AF detection [122].	PPG and MLAs.	Yes
	Wrist-worn Device (2017)	Discusses the development of a deep neural network to accurately classify AF using the PPG technique on the patient’s wrist [123].	PPG, accelerometer, and single-lead ECG.	Yes
Heart failure	Smart fitness trackers (2021)	Analyzes the physical activity of 70 patients with stable symptoms of heart failure through actigraphy [124].	Pedometer and accelerometer.	Yes
	Smart fitness trackers (2021)	Analyzes the current use of actigraphy in randomized controlled trials (RCTs) of patients with heart failure [125].	Pedometer and accelerometer.	Yes
	Wristband (2020)	Examines wristband technologies that can facilitate more accurate bedside testing, due to the difficulty of heart failure diagnosis by physical examination alone. The research concludes that wristbands can be used as a complementary tool in the bedside diagnostic evaluation and not as the only option [126].	PPG, accelerometer, and ECG.	Yes
	Smart watchband and chest patch-vest (2020)	Analyzes the use of sensors in wearable devices that measure biomedical variable signals (noninvasively) for use in patients suffering from heart failure [127].	PPG, accelerometer, and ECG.	Yes
	Chest vest (2020)	Evaluates the efficacy of early defibrillation with a WCD on the incidence of sudden cardiac death [128].	Cardioverter defibrillator.	Yes
	Patch (2020)	Evaluates remote and noninvasive monitoring and predicts rehospitalization for heart failure [129].	ECG and 3-axis accelerometer.	Yes
	Smart watchband and chest patch (2019)	Analyzes the applications and future of wearable devices in HF detection [48].	PPG, accelerometer, and ECG.	Yes
	Smartwatches–fitness trackers (2018)	Reviews current developments and challenges in portable monitoring technologies based on the PPG technique [37].	PPG.	Yes
	Smart watchband and the Multiparameter Intelligent Monitoring in Intensive Care II (MIMIC II) dataset (2017)	Reviews the performance (with respect to cost and diagnostic accuracy) of current health monitoring systems targeting patients with congestive heart failure (CHF) [130].	PPG and ECG.	Yes
	Smart watchband (2017)	Evaluates PA trackers to promote self-care in PA performed by patients with HF [131].	Pedometer and accelerometer.	Yes
	Vest (2017)	Proposes a safe WCD management strategy to avoid implantable cardioverter defibrillator (ICD) implantation in high-risk patients with advanced heart failure [132].	Cardioverter defibrillator.	Yes
Arrhythmia	Smart watchband (2019)	Discusses the feasibility of a regulatory framework to standardize and incorporate into medical practice the data generated by smart device management platforms [133].	PPG, accelerometer, and single-lead ECG.	Yes
	Electronic platform (2018)	Discusses the development of a portable medical system integrating a three-lead ECG sensor for real-time arrhythmia detection [134].	Texas Instruments TMS320C5515 and Raspberry Pi 3 Model B.	Yes
	Four databases from PhysioNet (2017)	Evaluates the precision of portable ECG devices for arrhythmia detection, achieving good results when compared to other previous studies [135].	ECG.	Yes
Ventricular fibrillation and sudden cardiac death (SCD)	Vest (2021)	Summarizes the literature on wearable cardioverter defibrillators (WCDs) [136].	Cardioverter-defibrillator.	Not applicable
	Vest (2018)	Reviews current data on WCD in newly diagnosed cardiomyopathy [137].	Cardioverter defibrillator.	Not applicable
Congestive heart failure (CHF)	Electrodes (2015)	The researchers discuss a clinical trial where they propose a technique to monitor the fluid status of patients with congestive heart failure in the hospital [138].	ECG, 3-axis accelerometer, and bioimpedance Z (BioZ).	Yes

**Table 4:** Noncommercial wearables with real-time monitoring capabilities.

CVD Type	Real-Time Monitoring	Distribution
Atrial fibrillation	Yes	95%
	No	5%
Heart failure	Yes	92%
	No	8%

**Table 5:** Sensors and technology implemented in noncommercial wearables for CVD monitoring.

CVD Type	Sensor/Technology Used	No. Used	%
Atrial fibrillation	ECG	10	38%
	PPG	9	35%
	Algorithm	5	19%
	Accelerometer	2	8%
Heart failure	Accelerometer	8	30%
	ECG	6	22%
	PPG	5	19%
	Cardioverter defibrillator	4	15%
	Pedometer	3	11%
	Bioimpedance Z	1	4%

## Data Availability

Not applicable.
